# Translational evidence for lithium-induced brain plasticity and neuroprotection in the treatment of neuropsychiatric disorders

**DOI:** 10.1038/s41398-021-01492-7

**Published:** 2021-07-05

**Authors:** Stefano Puglisi-Allegra, Stefano Ruggieri, Francesco Fornai

**Affiliations:** 1 IRCCS Neuromed, Via Atinense 18, 86077 Pozzilli (IS), Italy; 2 Human Anatomy, Department of Translational Research and New technologies in Medicine and Surgery, University of Pisa, Via Roma 55, 56126 Pisa (PI), Italy

**Keywords:** Addiction, Molecular neuroscience, Bipolar disorder

## Abstract

Increasing evidence indicates lithium (Li^+^) efficacy in neuropsychiatry, pointing to overlapping mechanisms that occur within distinct neuronal populations. In fact, the same pathway depending on which circuitry operates may fall in the psychiatric and/or neurological domains. Li^+^ restores both neurotransmission and brain structure unveiling that psychiatric and neurological disorders share common dysfunctional molecular and morphological mechanisms, which may involve distinct brain circuitries. Here an overview is provided concerning the therapeutic/neuroprotective effects of Li^+^ in different neuropsychiatric disorders to highlight common molecular mechanisms through which Li^+^ produces its mood-stabilizing effects and to what extent these overlap with plasticity in distinct brain circuitries. Li^+^ mood-stabilizing effects are evident in typical bipolar disorder (BD) characterized by a cyclic course of mania or hypomania followed by depressive episodes, while its efficacy is weaker in the opposite pattern. We focus here on neural adaptations that may underlie psychostimulant-induced psychotic development and to dissect, through the sensitization process, which features are shared in BD and other psychiatric disorders, including schizophrenia. The multiple functions of Li^+^ highlighted here prove its exceptional pharmacology, which may help to elucidate its mechanisms of action. These may serve as a guide toward a multi-drug strategy. We propose that the onset of sensitization in a specific BD subtype may predict the therapeutic efficacy of Li^+^. This model may help to infer in BD which molecular mechanisms are relevant to the therapeutic efficacy of Li^+^.

## Introduction

Lithium (Li^+^) remains the “gold standard” pharmacological agent for treating and preventing relapses in both type I and type II bipolar disorders (BDs). It is also effective in preventing suicidal behavior during major depression. Despite its strong efficacy, during the past years, Li^+^ was less and less administered due to toxicity and the need of monitoring serum concentrations to strictly maintain safe therapeutic doses [1–5]. However, alternative methods to administer low but effective therapeutic Li^+^ doses have been developed [6]. Besides its efficacy in treating and preventing symptoms of psychiatric disorders, Li^+^ produces neuroprotective effects on a wide range of neuronal populations that are involved in both behavioral and motor-related circuitries.

In fact, increasing evidence is under debate, indicating Li^+^ efficacy in neurodegenerative disorders, pointing to common intracellular mechanisms, which occur within distinct neuronal populations. This is the case of Alzheimer’s disease (AD), amyotrophic lateral sclerosis/frontotemporal dementia (ALS/FTD), and Parkinson’s disease (PD) [4, 7].

Knowledge of these mechanisms may shed light on the pathogenesis and neuropathology of different psychiatric and neurological disorders. The molecular mechanisms of action of Li^+^ involve classic pharmacologic targets, such as cell surface receptors or modulation of neurotransmitters, second messenger systems, enzyme cascades, and transcriptional factors [1, 8].

Here an overview is provided concerning the therapeutic/neuroprotective effects of Li^+^ in different neuropsychiatric disorders to highlight common molecular mechanisms through which Li^+^ produces its mood-stabilizing effects and to what extent this overlaps with neuroprotection in various brain circuitries. In fact, similar cell alterations depending on which circuits take place may fall either in the psychiatric and/or neurological domains.

## General overview of molecular mechanisms of Li^+^

Some effects of Li^+^ are related to the physical–chemical reversible competition with magnesium ion (Mg2^+^) within specific catalytic protein domains involved in substrate phosphorylation. Evidence shows Li^+^’s ability to inhibit Mg2^+^-dependent enzymes by displacing Mg2^+^ from its binding sites, thereby reducing enzyme stability and activity [9]. Thus, pharmacological actions of Li^+^ mostly depend on the reciprocal Li^+^/Mg2^+^ ratio [10]. These mechanisms of action add on the multifaceted pharmacology of Li^+^ [11], as well as its multiple targets.

The binding competition between Li+ and Mg2+ at substrate enzyme sites modulates the activity of several enzymes within intracellular pathways involved in biochemical effects, which are relevant both for neuropsychiatric and neurodegenerative disorders. These include inositol monophosphatase (IMPase), Akt/β-arrestin-2, and glycogen synthase kinase-3β (GSK-3β). There are two closely related forms of GSK-3, termed alpha (GSK-3α) and beta (GSK-3β), which are equivalently inhibited by Li^+^ [12]. Li^+^ inhibits GSK-3 by competing with Mg^2+^ for an essential binding site [13]. In addition to Mg^2+^ competition, Li^+^ inhibits GSK-3β activity by increasing its phosphorylation [14]. Li^+^ also decreases GSK-3β levels by inhibiting its transcription [15]. It is worth noting that, of all the kinases, GSK-3 influences the largest number of substrates [16]. Thus, Li^+^ has been estimated to act at several hundreds of GSK3-dependent substrates through the modulation of a number of GSK-3-dependent pathways. Since Li^+^ affects other Mg^2+^-dependent proteins, the occurrence of >3000 human proteins has been estimated that can be affected by Li^+^ [17]. This ability to act on a plethora of molecules, each one owning a functional relevance, makes Li^+^ a powerful pharmacological agent and a valuable tool in clinical and preclinical research.

During the past decades, evidence is provided showing that Li^+^ inhibits a number of phosphatases by acting as an uncompetitive and non-competitive inhibitor. Although the mechanisms of Li^+^-induced phosphatase inhibition are still not completely unveiled [18, 19], uncompetitive inhibition of IMPase and inositol polyphosphate 1-phosphatase (IPPase) has been recently proposed [19, 20]. Inhibition of IMPase and IPPase markedly reduces inositol triphosphate (IP3) levels, which in turn modulate many intracellular pathways known to be relevant for both neuropsychiatric and degenerative disorders, such as autophagy [1].

A number of neurotransmitters including dopamine (DA), norepinephrine, and serotonin act through G protein coupled receptors. Thus, the effects of Li^+^ on the inositol cycle may translate into altered receptor activity following monoamine stimulation, which in turn may dampen neurotransmitter efficacy, thus inducing synaptic stabilization [10].

Remarkably, Li^+^ also stimulates gene expression including brain-derived neurotrophic factor (BDNF) and vascular endothelial growth factor (VEGF). Low levels of BDNF during depressive, and manic phases in bipolar patients [21], have been reported to be reversed by Li^+^ treatment. In line with this, Li^+^ and BDNF plasma levels are inter-related [22]. Li^+^ also regulates inflammatory processes blunting the pro-inflammatory response. In detail, it decreases lipopolysaccharide-induced inflammation in glial cells [23] and reduces the production of interleukin-1 beta and tumor necrosis factor-alpha. The ability of Li^+^ to modulate inflammation is relevant to its pharmacological effects since the inflammatory processes play a crucial role both in neurodegeneration and mood disorders [24, 25].

**Fig. 1:**
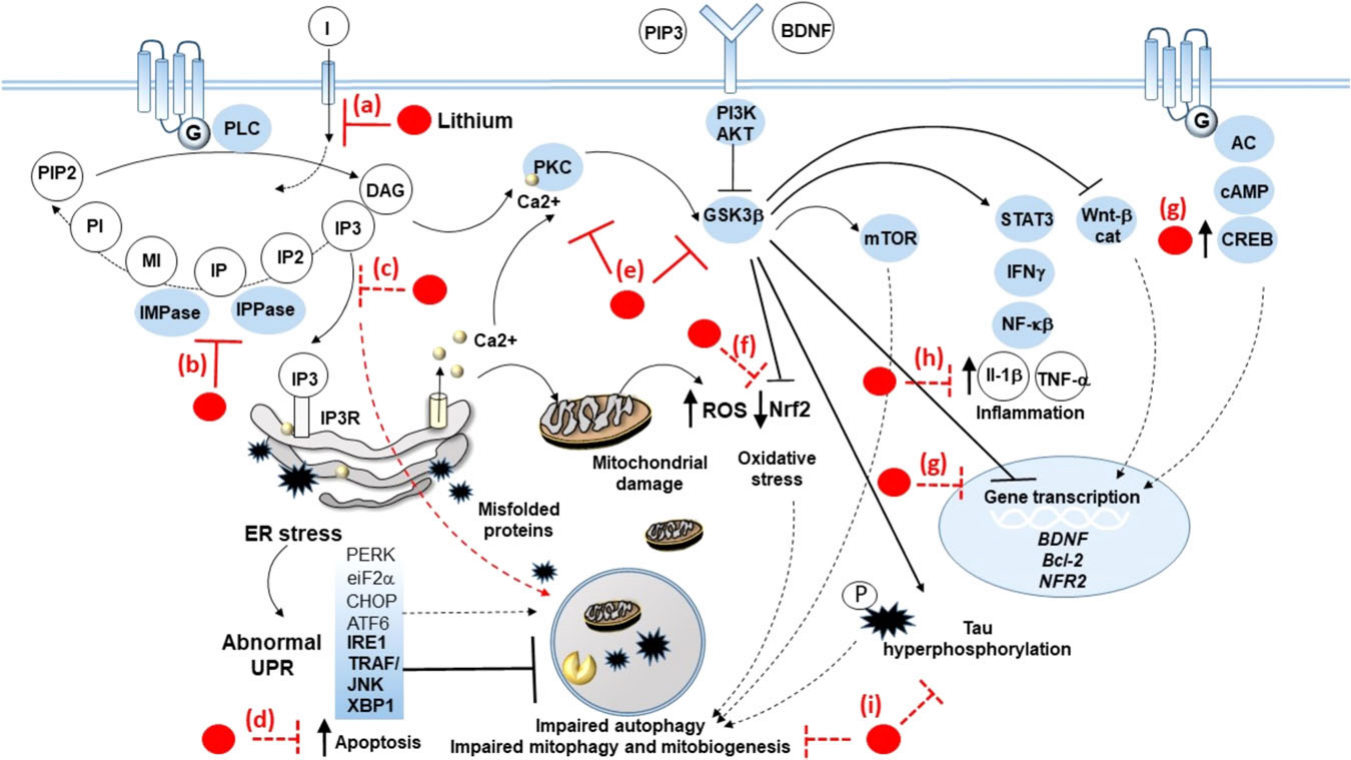
Detailed drawing of the fine molecular mechanisms of lithium on specific cell targets. Lithium inhibits the PI cycle, which is activated following stimulation of G-coupled neurotransmitter receptors (GPCRs). PLC mediates the hydrolysis of phosphotidylinositol 4,5-bisphosphate (PIP_2_) to the secondary messengers diacylglycerol (DAG) and inositol trisphosphate (IP_3_), which in turn activate downstream signaling pathways, including protein kinase C (PKC), and IP_3_ receptor (IP_3_R)/ER stress/unfolded protein response (UPR). In detail, lithium inhibits (a) the reuptake of inositol (I), as well as IMPase and IPPase (b), which results in overall depletion of IP_3_ (c). In this way, lithium prevents autophagy impairment and apoptosis that are bound to IP_3_R-related ER stress, massive Ca^2+^ release from the ER, abnormal UPR, as well as the accumulation of misfolded/unfolded proteins and damaged mitochondria (d). Abnormal UPR consists, for instance, of upregulation of TRAF/JNK and XBP1, which fosters apoptotic events while impairing autophagy. At the same time, through inhibition of PKC and GSK3β (e), lithium inhibits potentially deleterious effects triggered downstream of these pathways. These include (f) oxidative stress due to accumulation of reactive oxygen species (ROS) leaked from damaged mitochondria and downregulation of the nuclear factor erythroid 2 (NFE2)-related factor 2 (NRF2), which, besides its antioxidant effects, is also related to mitophagy and mitochondriogenesis; (g) impaired transcription of neurotrophic, neuroprotective, and antioxidant genes, such as BDNF, VEFG, Bcl-2, and NRF2, which is instead reinstated by lithium via both GSK3β inhibition and activation of the CREB transcription factor placed downstream of GPCRs; (h) production of pro-inflammatory cytokines underlying activation of the STAT/interferon gamma (INFγ)/nuclear factor kappa-light-chain enhancer of activated B cells (NF-kβ) pathway; and (i) accumulation of hyperphosphorylated tau, which is bound to cytoskeletal alterations and autophagy impairment.

Li^+^ targets unfolded protein response (UPR)-related events, such as endoplasmic reticulum (ER) stress, excitotoxicity, and autophagy dysfunction either at the synapses or within cell bodies. This is expected to induce neuroplasticity, which affects behavior and motor activity, through overlapping mechanisms in different brain areas (Fig. 1).

UPR activation induces autophagy [26, 27], which counteracts ER stress via degradation of protein aggregates and organelles, including damaged mitochondria, nuclear membrane, and ER. Moreover, Li^+^ rescues autophagy failure, which is often reported in neurodegenerative disorders, including PD, AD, tauopathies, ALS, and Huntington disease [7, 12, 25, 28–42]. Similarly, autophagy is often reported to be dysregulated in a number of psychiatric disorders.

Autophagy is a phylogenetically conserved eukaryotic cell-clearing system that plays a seminal role in cell homeostasis [36]. It is distinguished into macro-autophagy, micro-autophagy, and chaperone-mediated autophagy, which all promote lysosome-dependent substrate degradation [37]. Autophagy can be induced by a number of cascades, among which the one controlled by the mammalian target of rapamycin complex1 (mTORC1) plays a relevant role. The mTOR complex represents a downstream substrate of the phosphoinositide-3 kinase/phosphatase and tensin homolog/Akt pathway.

Activation of 5′ AMP-activated protein kinase and inhibition of GSK3-β [8, 41] are other well-known pathways controlling autophagy initiation.

Besides degrading oxidized/misfolded proteins and damaged organelles, autophagy modulates key cell functions ranging from neural tube and synapse development to neurotransmitter release and synaptic plasticity, as well as neuroinflammation and immunity [4, 43].

Although Li^+^ inhibits GSK-3β, which reduces autophagy via mTOR activation, the prevalent effect of Li^+^ consists in mTOR-independent autophagy activation since Li^+^ strongly inhibits IMPase [7, 44]. In line with this, inositol depletion has mood-stabilizing effects [1, 42], while in tauopathies Li^+^ may play a dual protective role by inhibiting GSK-3β [45], producing strong autophagy-independent neuroprotective effects and counteracting tau accumulation through autophagy activation ([46], Fig. 1).

Autophagy activation is expected to clear damaged mitochondria (mitophagy), and by a concomitant increase in mitochondriogenesis, this speeds up mitochondrial turnover, which improves mitochondrial function. Li^+^-induced mitochondriogenesis and mitophagy occur both in endothelial cells [47] and specifically within various neuronal types [27, 34].

Such an effect, which is overtly crucial in neurodegeneration, also applies to mood disorders since mitochondrial dysfunction is reported in bipolar patients [48, 49].

Thus, even considering Li+ strong therapeutic effects on mood disorders, it is likely that autophagy takes a center stage in its antimanic and antidepressant action. This is not surprising when considering that a number of autophagy-inducing drugs, which are on-label with other therapeutic indications, possess mood-stabilizing effects (i.e., valproate, rapamycin, [46]). Similarly, the specific efficacy on mood disorders of a variety of psychotropic drugs may partly relate to autophagy-inducing effects [50–52].

## Lithium in neurodegenerative and psychiatric disorders

### Neurodegenerative disorders: AD and PD and ALS/FTD

A growing body of evidence points to the neuroprotective effects of Li^+^. Subjects with BD administered with long-term Li^+^ have a lower risk to develop dementia including AD [53, 54]. A meta-analysis study [55] shows that Li^+^ treatment significantly decreases cognitive decline as compared with placebo, thus indicating that Li^+^ may be beneficial in promoting cognitive performance in subjects with mild cognitive impairment (MCI) and AD.

Li^+^ may act at multiple steps within the biochemical cascades involved in the onset and progression of AD (Fig. 1). For instance, GSK-3β inhibition may counteract the pathological increased enzyme activity occurring in patients affected by MCI and AD [56]. GSK-3β is involved in amyloidogenesis and tau phosphorylation, which at the preclinical level are inhibited by Li^+^ administration [57]. Again, Li^+^-induced autophagy may counteract autophagy suppression, which is expected in AD patients due to a progressive increase in mTOR activity during the disease course [35, 58]. Moreover, Li^+^ promotes the synthesis and release of neurotrophic factors, in particular BDNF and VEGF whose increased availability protects neurons against neurotoxic insults, stimulates hippocampal neurogenesis, and increases synaptic plasticity [2]. Indeed, BDNF polymorphisms were reported to moderate Aβ-related cognitive decline in preclinical AD [59], and BDNF reduces Aβ in the brain [60]. However, a short half-life and inability to cross the blood–brain barrier impair the therapeutic potential of BDNF [61].

Such a limitation is overcome by small molecules like BDNF mimetic compounds that are able to protect primary neurons from Aβ-induced toxicity and to promote synaptogenesis [62]. Moreover, Li^+^ is able to effectively activate the molecular pathway increasing BDNF synthesis [10] It is worth noting that animal models play a key role in AD research also fostering clinical studies in patients to assess Li^+^ efficacy in contrasting Aβ and tau pathology [63].

ALS is a motor neuron disease, belonging to a group of neurological disorders that selectively affect motor neurons controlling voluntary muscles of the body. ALS can be classified as familial or sporadic, depending on whether or not there is a family history of the disease. Although there is no consensus on the familial definition, a number of genes are considered to cause the disease.

Motor symptoms improvement by Li+ was reported during the past two decades. Thus, in a clinical trial, Li^+^ treatment for 15 months was shown to be safe and significantly associated with a slower rate of disease progression and death [28]. Substantial neuroprotection accompanied by delayed disease onset and increased life span was shown in G93A murine model [28]. It should be pointed out that several ALS genetic murine models have been developed [64]. Although of huge utility in preclinical research, they are partially representative of the pathology and the efficacy of lithium in these models deserves to be further investigated [65].

Daily doses of Li^+^, leading to plasma levels ranging from 0.4 to 0.8 mM, delay disease progression in a small group of ALS patients [28]. This was further validated in a stratified study on ALS patients carrying the *UNC13A* variant where Li^+^ doubles survival time [29], while in a heterogeneous ALS population these protective effects of Li^+^ are debatable. Thus, it is clear how Li^+^ affects multiple targets, all of which are likely to contribute to the improvement of ALS, such as autophagy that involves Li^+^ inhibitory action on GSK-3 and IP_3_ turnover or suppression of glial cell activation in the spinal cord [4].

Li^+^ induces autophagy to counteract ER stress and altered UPR and rescues autophagy failure occurring in both ALS/FTD and BD [7, 28, 34, 35, 49, 66–72]. Noteworthy, UPR markers (p-eIF2a, GRP78, GRP94, XBP1, and CHOP) have been shown to predict Li^+^ responsiveness in bipolar patients [70].

Li^+^-responsive psychiatric disorders, such as BD, depression, and anxiety, may often precede ALS/FTD, and patients with psychiatric disorders receiving regular Li^+^ treatment have a reduced prevalence of ALS and dementia [73].

This evidence shows once more that neurodegenerative diseases and affective disorders may share common neural mechanisms into which Li^+^ acts as therapeutic, strongly suggesting that these mechanisms are crucial in etiology.

Li^+^ efficacy in PD has been poorly studied and results indicate that it deserves substantial clinical trials to ascertain promising neuroprotection as suggested by experimental models.

In a parkin mutant transgenic mouse, low doses of Li^+^ prevent motor impairment as well as dopaminergic striatal degeneration, parkin-induced striatal astrogliosis, and microglial activation. These results further validate Li^+^ as a potential therapy for PD [74]. At first glance, this may sound odd since Li^+^ by impeding sensitization produced by DA [75] is expected to attenuate the efficacy of the long-term L-DOPA response, which represents an important part of L-DOPA symptomatic effect. Thus, a symptomatic interference of Li^+^ with PD may hide the disease-modifying effect produced by Li^+^ acting on autophagy-dependent ongoing degenerative steps [7, 69].

Alpha-synuclein, a major substrate of autophagy, accumulates in Lewy bodies, which are mostly found within spared dopaminergic neurons of the substantia nigra pars compacta [76], as well as within extra-nigral neuronal populations [77].

Moreover, genetic ablation of *Atg7* specifically within DA neurons fully reproduces PD pathology, including the formation of alpha-synuclein-stained Lewy bodies. Evidence that points to a key role of autophagy in DA-related disorders [78] and Li^+^ as a potential therapy for PD has been provided [4].

### Psychiatric disorders: BD, and major depressive disorder (MDD), and schizophrenia

Li^+^ mood-stabilizing effects are evident in typical BDs characterized by a cyclic course of mania or hypomania followed by depressive episodes, while efficacy is weaker in the opposite pattern [5]. In fact, Li^+^ is less effective in bipolar depression, in rapid cycling, in psychotic syndromes, in drug abuse, and in depression-prone cases [5]. Moreover, the effects produced by Li^+^ in major depression are not as significant as those produced in cycling BD, as we report briefly below.

Li^+^ has been proposed for treating MDD since the last decades of the past century [79, 80] as an alternative pharmacotherapy for those patients who were refractory to antidepressants. At present, no clear evidence is available on the efficacy of Li^+^ monotherapy compared either with placebo or antidepressants in acute, unipolar, major depressive episodes [78]. However, a number of studies have reported the beneficial effects of Li^+^, even when it is administered as an adjunct to antidepressant treatment [81, 82].

Li^+^ has been consistently reported to be effective in reducing the suicide risk both in unipolar and bipolar patients [83], possibly acting on GABA [67] to modulate aggression and impulsivity [84–86] via autophagy [87].

Li^+^ is not effective in schizophrenia, as documented by literature from the past decades (see [86] for a review). Albeit Li^+^ is co-administered with specific antipsychotics to relieve some side effects, this does not imply any efficacy of Li^+^ to treat schizophrenia [88, 89]. This may sound unexpected since schizophrenia and BD share some functional and symptomatic features. Indeed, this discrepancy in therapeutics confirms what was originally indicated by Emil Kraepelin who in 1899 distinguished “dementia praecox” (further called schizophrenia) from “manisch-depressive irreisen,” “manic-depressive insanity,” and manic-depressive disorder, nowadays named BD. Such a distinction is currently maintained despite some conflicting hypotheses [90].

We do not wish to deal with such a nosographic issue; we rather want to emphasize how the discrepant effects of Li^+^ in these disorders may rely on distinct molecular mechanisms operating in schizophrenia compared with BDs, owing to distinct molecular targets.

This is a crucial point since schizophrenia and BD share some behavioral alterations and neurotransmitter changes (e.g., DA and glutamate). Moreover, genetic correlations and common genes between these disorders have been shown [91–93].

These similarities suggest that these disorders do possess some overlapping steps in their molecular mechanisms. However, the simple fact that Li^+^ is poorly or no effective in schizophrenia urges to investigate which Li^+^-dependent or Li^+^-independent molecular pathways exist in BDs compared with schizophrenia, respectively [94, 95].

Why then Li+ is effective in BD and possesses a poor efficacy if any, in schizophrenia? We will discuss such a difference by pointing out the cycling nature of BD in contrast with a progressive course of schizophrenia.

It is important to note that the molecular pathways that are implicated in the sensitization phenomenon are crucial in schizophrenia, in BD, as well as in psychotogenic effects of abused psychostimulants. In fact, the cycling nature of BD may be key in the specific pattern of sensitization, which develops in such a disorder, contrasting with a progressive course, which otherwise takes place in schizophrenia.

Li^+^ is effective in suppressing or delaying psychostimulant-induced sensitization [96] by acting on some common mechanisms also shared by schizophrenia and BD.

Although both disorders share sensitization mechanisms, only BD is sensitive to Li^+^, which remains in search of an explanation. We suggest that a sensitization process exists in both disorders although the time course of such a sensitization diverges, which explains the different outcome of a similar pharmacological manipulation.

Sensitization is a process through which repeated intermittent exposure to a given stimulus, such as stress, trauma, or psychostimulants, leads to enhanced behavioral responses to subsequent exposure [97–99].

The presence of behavioral sensitization in patients with BD has been proposed to explain relapses and the progression of behavioral dysfunction [97, 100].

The effects of psychostimulants are relevant to study the molecular mechanisms that operate in the sensitization process, which takes place in psychiatric disorders and is shared by schizophrenia and BD. In fact, when psychostimulants are chronically abused, psychotic or manic symptoms may occur, which further provides witness for a promiscuous overlap between molecular mechanisms operating in these disorders.

Therefore, we focus here on the effects of psychostimulants on neural adaptations underlying drug abuse and psychotic development, to highlight, through the sensitization process, which features are shared or not between schizophrenia and BD.

### Li^+^, dopamine-related sensitization, and cell-clearing systems

Amphetamine (AMPH) repeated administration induces behavioral sensitization revealing a suited manic-like preclinical model [101–103]. Translational validity of sensitization to psychostimulant psychological stress [97, 104–106] is also supported by the overlapping of their neural effects, especially on dopaminergic transmission. This may also explain why AMPH induces manic symptoms in both healthy volunteers and BD subjects [107].

Chronic cocaine in preclinical model produces behavioral sensitization and decreases β-catenin levels in the prefrontal cortex, amygdala, and dorsal striatum. Accordingly, GSK-3β activity levels in these areas are increased [108]. Li^+^ treatment rescues β-catenin levels and blocks cocaine-induced sensitization [108]. These results are consistent with Li^+^-induced GSK-3β inhibition and the following increase in β-catenin levels. However, contrasting results show increased β-catenin levels within nucleus accumbens that parallels the expression of cocaine sensitization, both phenomena being rescued by Li^+^ administration [109, 110], thus casting a scenario in which Li+ effects appear contradictory. Possible involvement of neurotransmitters has been advocated to explain these differences and a role of neural networks, in which reciprocal influences between brain areas may affect them differently [109].

Methamphetamine (METH), as well as other psychostimulants, commonly produces psychoses with positive symptoms similar to those of schizophrenia. Such an effect makes METH use/abuse to be commonly considered as an experimental model of schizophrenia. High pre-synaptic DA synthesis and release are peculiar of schizophrenia [111–113]; likewise, the psychostimulant effects of METH rely on increased DA synthesis and massive DA release within limbic and dorsal striatal areas, as well as abnormal stimulation of postsynaptic DA receptors (DARs), mainly the D1 subtype (D1Rs) [114, 115]. On the other hand, schizophrenic patients are oversensitive and overresponsive to AMPHs [98, 111].

METH dysregulates a number of susceptibility genes for schizophrenia, such as DISC1, NRG1/ErbB4, and CRMP2, which are known to be involved in the regulation of presynaptic DA release or post synaptic D1R-related cascades. Noteworthy, they all converge on mTOR signaling, thus abnormal stimulation of D1Rs activates significantly mTOR, which inhibits the autophagy machinery [116]. This suggests that altered mTOR and impaired autophagy pathway represent a common hub between psychostimulant-induced neuroplasticity and schizophrenia.

METH impairs the ubiquitin proteasome system (UPS) activity, which is largely dependent on DA [117–122]. Proteasome regulates DA presynaptic release and receptors and postsynaptic DARs. Activation of DAD2 receptor subtype (D2Rs) contributes to the expression of DA-dependent METH-induced behavioral alterations also via a signaling complex composed of β-arrestin 2 (β-arr2), AKT, and protein phosphatase-2A (PP2A), which activates GSK-3β [123–126].

**Fig. 2:**
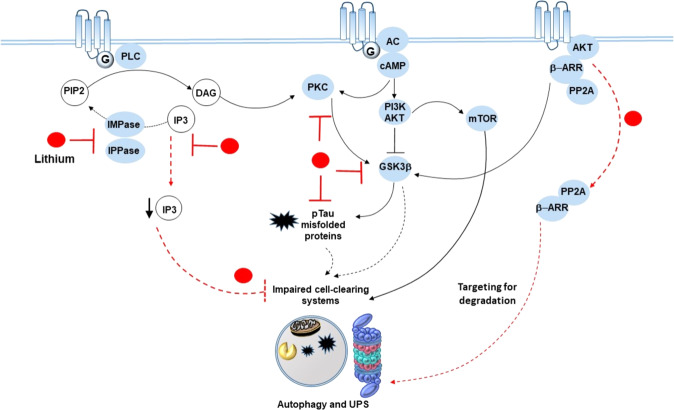
Lithium, amphetamine-related sensitization, and cell-clearing systems. Lithium inhibits IMPase and IPPase, as well as PKC and GSK3β, which are triggered downstream of dopamine GPCRs. This results in reduced production and phospho-tau/misfolded proteins and the rescue of cell-clearing systems, which are impaired by mTOR hyperactivation placed downstream of D1 DA receptors and/or by abnormal amounts of phospho-tau. At the same time, lithium disassembles the GSK3-β-activating proteins β-ARR and PP2A from the complex they form with GPCRs/AKT meanwhile targeting them for degradation by the cell-clearing systems. In this way, lithium adjusts dopamine imbalances that occur following psychostimulant intake/administration and abnormal activation of GPCRs and related downstream signaling pathways.

Li^+^ is a direct inhibitor of GSK-3 and also inhibits GSK-3 activity in the cell through an indirect mechanism that involves Akt activation. Li^+^’s ability to disrupt β-arr2-mediated Akt/GSK-3 signaling contributes to suppressing the behavioral effects of enhanced DA transmission (Fig. 2, [123–127]).

D2Rs activate a signaling complex composed of β-arr2, Akt, and PP2A, which activates GSK-3β [123–126, 128, 129] and fosters DA-dependent behavioral changes induced by METH. Li^+^ antagonizes DA-related behavioral sensitization through inhibition of GSK-3β (Fig. 2, [123–127]). All components of the complex that activates GSK3β, including β-arr2, AKT, and PP2A, are UPS substrates, whose inhibition, similarly to METH and DA, activates GSK-3β, while, conversely, GSK-3β inhibition protects from UPS inhibition-induced toxicity (see [122] for a review).

Furthermore, Li^+^ can blunt DA transmission through IP_3_ and protein kinase C (PKC) placed downstream of DA1Rs or D1/D2 heterodimers [130, 131].

Based on this body of evidence we suggest that the occurrence of sensitization in a subtype of BD may be relevant to the therapeutic outcome of Li^+^ in the very same BD phenotype. This may allow inferring on which molecular mechanisms are relevant to the therapeutic effects of lithium in BD.

Li^+^ is notoriously highly effective in BD1 compared to the hypomanic phase that characterizes the type 2 disorder (BD2). Although the usefulness of such a distinction between BD1 and BD2 is not unanimously shared [132], it represents an important element for understanding the action of Li^+^, together with blunted Li^+^ efficacy on BD depression compared with mania.

Evidence for Li^+^ therapeutic efficacy in BD1 clearly indicates that its action is intimately linked to the peculiar feature of the disorder, namely cyclicity. In fact, it is legitimate to infer that during the phase in which mania progressively rises (“mania rising”), as expressed by behavioral phenotype, Li^+^ has a sort of higher “affinity” [133] for neural events spurring the “manic rise.”

**Fig. 3:**
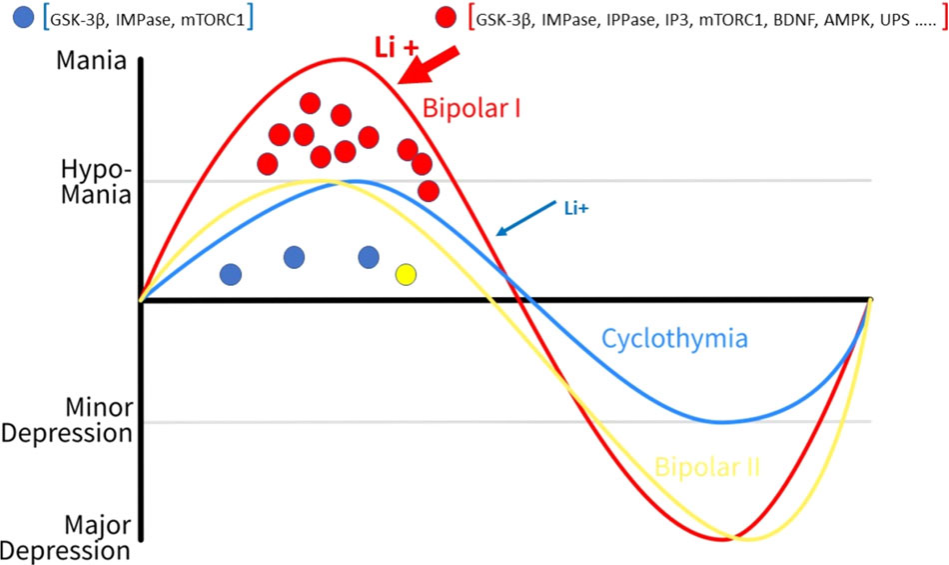
Neural molecular substrates of lithium determining its differential affinity and efficacy for BD1 vs BD2. Circles represent neural molecular substrates of Li^+^. Red circles represent the most abundant neural targets of Li^+^ underlying its therapeutic efficacy in mania (BD1); blue and yellow circles indicate a progressively decreasing number of neural molecular substrates of Li^+^ in minor and major depressive disorders (cyclothymia and BD2).

The factors implicated in this affinity should be sought in the “dynamic” status of systems where a number of molecular machineries would be “on fire” up to the peak. In fact, the syndrome “evolves” progressively towards the full expression of the manic phase (Fig. 3). The molecular factors of this “dynamic” condition are a serious candidate to unveil which mechanisms allow lithium to be most effective in this phase of BD1.

As reported above, sensitization is driven by molecular pathways linked to DA transmission, which owns a crucial role in BD [134]. Brain DA during the “mania rising” phase is dramatic and produces sensitization, as clearly indicated by behavioral, neural, and pharmacological evidence ([134], for review).

Importantly, sensitization progresses differently in different disorders. Thus, as evident also from behavioral changes, in BD1 the peak of mania is reached in the temporal order of weeks. Psychostimulant-induced sensitization falls into a similar (even shorter) temporal window. In schizophrenia, the process, also referred to as endogenous sensitization [135], develops over a longer period.

In each disease, two stages can be distinguished during the progression: one leads to a change in neural activity up to “peak,” while the other stabilizes a steady state of modified activity once the peak is over. In fact, in BD, the mania phase recovers toward an adjustment that leads to a subsequent phase (euthymia or depression). Molecular mechanisms are mostly overlapping in these three disease conditions; however, since they do occur according to a different time course, they are likely to engage diverse and specific molecular pathways. Such a different progression may explain the contingent efficacy of Li^+^ based on the time window when it is administered.

On the sidelines, let us consider briefly that once the manic phase is over some of the molecular pathways involved in “mania rising” are also involved in the phase of depression [126], possibly to counterbalance the sensitization adjustments through reverse mechanisms. This may exceed the adjustment which is needed, thus producing an outcome, which is “opposite” to mania. It can be hypothesized that Li^+^’s action on these pathways antagonizes depressive mood; nonetheless, the biological substrate to which Li^+^ binds is not abundant enough to allow stochastic Li^+^ binding to be adequate in a short time interval. This would not allow Li^+^ to fully express its pharmacological activity. This view is supported by preclinical evidence showing that AMPH withdrawal after a repeated administration induces depressive-like behaviors [99, 136]. However, Li^+^ could maintain a blunted depressive mood by balancing neural mechanisms underlying motivational systems, which are regulated by DA transmission [106, 134, 137].

This progression brings to mind a sentence of an esteemed scholar of BD, Athanasios Koukopoulos, “Mania is the fire and depression its ash” [138, 139]. This leads to consider a crucial effect of Li^+^ action, namely the ability, once mania phase is over (corrected), to maintain a functional balance that leads to “stabilization.”

It is important to consider that the therapeutic efficacy (although in a significant percentage of patients) is relevant in BD1, in many cases of psychostimulant sensitization, although it is practically absent in the case of schizophrenia, when its efficacy is minimal in rapid cycling and in BD2.

Major differences in sensitization between these disorders ground on time course and intensity (severity) of altered biochemistry, drug sensitivity, and disturbed behavior. The clearest differences in the time course occur between BD1 and schizophrenia as well as between slowly vs rapid-cycling BD1, while the most relevant difference in symptom intensity of the manic phenotype is between BD1 and BD2 or “mixed” states [140].

Importantly, a substantial increase in DA transmission following acute AMPH challenge was observed in patients with schizophrenia compared with healthy control subjects. These effects were evident in patients being at the onset of the disease and who were never previously exposed to neuroleptics or in patients experiencing an episode of illness exacerbation, but not during a remission phase [111]. This evidence indicates that DA hyperactivity is present in the schizophrenic patient at the onset of symptoms, in the relapse phases, and, possibly, during the prodromal period.

This suggests that marked Li^+^ efficacy in BD1 is due to a massive DA activation, subsequent sensitization, and molecular mechanisms that are activated far in excess compared with other disorders. The molecular substrates activated by the DA overload could offer Li^+^ a privileged “hooking” that would result in effective regulatory action and, in turn, effective therapy.

## Discussion, limitations, and conclusions

In the present manuscript, we focused on the therapeutic effects of Li^+^ to highlight common mechanisms of action in different neuropsychiatric disorders. These mechanisms provide valuable information to help to understand the etiology of disorders on which they are involved and point to possible therapeutic developments. Li^+^ modulates a number of biochemical pathways in the brain involved in neuroplasticity and neuroprotection.

Li^+^ neuroprotective action relies on the modulation of several intracellular pathways involved in ER stress, mitochondrial function, Ca^2+^ toxicity, UPR, and autophagy. The effects on the autophagy machinery remain the key molecular mechanisms to explain the protective effects of Li^+^ for neurodegenerative diseases, which indicates how Li^+^ exploits similar molecular machinery to modify the course of different disorders belonging to different domains.

Such molecular machinery may have a neuroprotective and neurotrophic action on AD and other degenerative dementias. In fact, upregulation of GSK-3β activity, which is inhibited by Li^+^, occurs in AD.

A failure of autophagy-dependent handling of misfolded proteins impedes the clearance of these substrates that accumulate within the cell. Therefore, a common pathogenesis underlying neurodegenerative disorders has been linked to autophagy inhibition due to mTOR activation. Common mechanisms are involved in Li^+^ protective effects in neurodegenerative ALS/FTD disease as shown by significant improvement of motor function by treatment with Li^+^ during the past two decades in clinical and preclinical studies.

Li^+^ property to modulate autophagy offers potential therapeutic strategies for the treatment of neuropsychiatric disorders and emphasizes a crossroad linking autophagy, neurodegenerative disorders, and mood stabilization (antimanic activity). Remarkably, the molecular events in the progression of mania concern neuroplastic mechanisms implicated in neurotransmission as well as mechanisms of neurodegeneration.

Sensitization being a neuroplastic process that is crucial in the pathogenesis of main affective disorders, even slight differences in the sensitization process between these disorders may help to identify why some of them are sensitive to Li^+^, which is the case of BD1. Sensitization by psychostimulants is driven by molecular pathways linked to DA transmission and points to a number of mechanisms involved in psychopathology, most also crucial in neurodegenerative disorders.

Li^+^, as it is commonly acknowledged, has multifunctional power, in the sense that it impinges on different molecular pathways. In the case of the manic phase of BD1, the many pathways and the different mechanisms on which Li^+^ acts can constitute a multifunctional complex fostering its activity (Fig. 1).

During “mania rising”, sensitization at intracellular level recruits a number of molecular pathways that are matched by the same number of substrates binding Li^+^.

Sensitization process develops through dysregulation of various DAR subtypes, mainly D1, D2, and D3, at the presynaptic and/or postsynaptic level [126], including the involvement of the DA transporter. It should be borne in mind that, although the meso-accumbens and meso-striatal pathways are markedly affected, cortical structures that regulate subcortical areas in integrated systems have a fundamental role. Thus, due to sensitization, monoaminergic systems in the medial prefrontal cortex undergo receptor or metabolic adaptations that are not paralleled by those taking place within subcortical areas [105, 106, 132, 141–143]. Therefore, molecular pathways implicated in network alterations are likely to provide further substrates for Li^+^ efficacy.

Noteworthy, some apparent contradictory actions of Li^+^ on molecular and/or system levels may witness a simultaneous activity on those different systems or subsystems [14, 144].

The unique therapeutic efficacy of Li^+^ in mania may also be grounded on the ability of the ion to act on a wide number of molecular pathways and factors regulating neurotransmission [145], neuroplasticity, and neuroprotection. It can be assumed that functional alterations in “mania rising” bring into play molecular events on which Li^+^ engages to counteract the move away from steady state (homeostasis). Noteworthy, Li^+^ has been shown to be “attracted” from brain neurons of BD patients and not from those of healthy subjects [133], demonstrating an “affinity” of the ion for dysfunctional neural molecular substrates. This affinity can also be modulated by genetic factors that are obviously implicated in individual differences in therapeutic efficacy. In rapid-cycling disorders, the symptoms of the two states in short times rules out a cyclicity such that characterizing the progress of BD1. It is tempting to hypothesize that a lower efficacy of Li^+^ in these disorders is due to a lack of cyclicity and a longer time course and more intense biochemical alterations. This sharply contrasts with what occurs in BD1, and it is supposed to prevent a substantial substrate being hooked by Li^+^ for its effective therapeutic action.

The multiple functions of Li^+^ prove its exceptional pharmacology, which may help to elucidate its mechanisms of action. Li^+^ actions may serve as a guide toward a multi-drug strategy and an improved development of a more effective therapeutic approach.

Our view is not devoid of limitations. We considered molecular mechanisms common to a number of disorders highlighting their role in neurodegeneration and neuroprotection, addressing autophagy machinery as a relevant mechanism for neural regulation and dysregulation in both neurodegenerative and psychiatric disorders. A potential limitation consists in the lack of a deep analysis on how all these mechanisms lead to neurodegeneration, in different neuronal populations within different brain areas. In fact, a sharp brain tissue alteration occurs in neurodegenerative disorders as well as in schizophrenia and BD [146].

Another limitation, inherently analyzed in the previous text, is the lack of an extensive analysis on diverse cell populations including various interactions between neural networks as well as neurons and glia [147, 148] These include a specific analysis of neurons hosted within specific brain areas being part of complex networks whose connections can favor or dampen neurodegenerative processes [131] Such a lack of diversified analysis may be crucial when referring to the sensitization process that takes a center stage in the scenario provided here. In fact, purposefully and maybe in a biased way, we focused on DA transmission dysregulation, which led to emphasize different types of DARs whose alteration can produce different effects depending on whether they are presynaptic or postsynaptic. Moreover, dopaminergic neurons receive connections from a number of neurons in reciprocal DA cortical–subcortical networks, with different neurotransmitters and neuromodulators, which in turn may modulate neurodegenerative processes. These are just examples that show how to understand the processes addressed here; a wider system perspective is required in humans and preclinical models to reach relevant translational results.

Indeed, imaging and molecular analysis methods may facilitate the discovery of molecular and neurobehavioral biomarkers. In this perspective, Li^+^ proves an extraordinary tool for understanding pathogenic mechanisms and for developing effective therapies.

At the risk of appearing too much “conservative,” we do reaffirm the uniqueness of Li^+^ both as a therapeutic agent and as an extraordinary tool in neuroscience research. This is grounded right on its ability to foster, at the same time, neurotransmission and neuroprotection. These properties also unveil that psychiatric and neurological disorders share common dysfunctional molecular mechanisms that can be equally relieved both concerning symptomatic cure and disease-modifying events.
